# Plasmonic Metasensors Based on 2D Hybrid Atomically Thin Perovskite Nanomaterials

**DOI:** 10.3390/nano10071289

**Published:** 2020-06-30

**Authors:** Shuwen Zeng, Guozhen Liang, Alexandre Gheno, Sylvain Vedraine, Bernard Ratier, Ho-Pui Ho, Nanfang Yu

**Affiliations:** 1 XLIM Research Institute, UMR 7252 CNRS/University of Limoges, 123 Avenue Albert Thomas, 87060 Limoges, France; alexandre.gheno@xlim.fr (A.G.); sylvain.vedraine@xlim.fr (S.V.); bernard.ratier@xlim.fr (B.R.); 2 Department of Applied Physics and Applied Mathematics, Columbia University, New York, NY 10027, USA; gl2585.columbia@gmail.com; 3 Department of Biomedical Engineering, The Chinese University of Hong Kong, Shatin, N.T., Hong Kong 999077, Hong Kong; aaron.ho@cuhk.edu.hk

**Keywords:** surface plasmon, optical sensor, 2D materials, plasmonic sensing, differential phase

## Abstract

In this work, we have designed highly sensitive plasmonic metasensors based on atomically thin perovskite nanomaterials with a detection limit up to 10^−10^ refractive index units (RIU) for the target sample solutions. More importantly, we have improved phase singularity detection with the Goos–Hänchen (GH) effect. The GH shift is known to be closely related to optical phase signal changes; it is much more sensitive and sharp than the phase signal in the plasmonic condition, while the experimental measurement setup is much more compact than that of the commonly used interferometer scheme to exact the phase signals. Here, we have demonstrated that plasmonic sensitivity can reach a record-high value of 1.2862 × 10^9^ µm/RIU with the optimum configurations for the plasmonic metasensors. The phase singularity-induced GH shift is more than three orders of magnitude larger than those achievable in other metamaterial schemes, including Ag/TiO_2_ hyperbolic multilayer metamaterials (HMMs), metal–insulator–metal (MIM) multilayer waveguides with plasmon-induced transparency (PIT), and metasurface devices with a large phase gradient. GH sensitivity has been improved by more than 10^6^ times with the atomically thin perovskite metasurfaces (1.2862 × 10^9^ µm/RIU) than those without (918.9167 µm/RIU). The atomically thin perovskite nanomaterials with high absorption rates enable precise tuning of the depth of the plasmonic resonance dip. As such, one can optimize the structure to reach near zero-reflection at the resonance angle and the associated sharp phase singularity, which leads to a strongly enhanced GH lateral shift at the sensor interface. By integrating the 2D perovskite nanolayer into a metasurface structure, a strong localized electric field enhancement can be realized and GH sensitivity was further improved to 1.5458 × 10^9^ µm/RIU. We believe that this enhanced electric field together with the significantly improved GH shift would enable single molecular or even submolecular detection for hard-to-identify chemical and biological markers, including single nucleotide mismatch in the DNA sequence, toxic heavy metal ions, and tumor necrosis factor-α (TNFα).

## 1. Introduction

In the past decade, biosensors have provided an effective way for improving quality of life [1,2,3]. Biosensors are usually based on systems that can detect electronic or optical signals in terms of the concentrations of biological molecules [4,5,6,7,8], where molecular interactions can be monitored by the signal change. Useful applications include DNA analysis, glucose concentration tests in human blood, and sensing of toxins in the water, food, and atmosphere [9,10,11]. According to a recent report [12], the sales market for biosensors has reached $16.34 billion worldwide in 2016 and is expected to almost double by $34.3 billion in 2025. Current challenges for biosensors are to improve their detection sensitivity and reduce size and operation cost. Handheld biosensors with cost-effective sensing substrates are highly desired for medical and environmental tests [13]. At the beginning of market development, biosensing products typically require time-consuming and costly labelling processes, where a receptor is used with a fluorescent molecule bound with the target analytes. To achieve simple and fast detection, various label-free biosensing technologies [14,15,16,17] have been investigated and demonstrated. 

Plasmonic sensors or surface plasmon resonance (SPR) sensors are one of the most commonly-used optical sensing devices for real-time monitoring of chemical and biomolecular interactions [18,19,20,21,22,23]. The resonance occurs at an interface between a metal and a dielectric, and is a result of collective electron charge oscillations in the metal coupled to an interfacial electromagnetic wave. SPR is very sensitive to the surrounding environment, a property that is utilized in real-time and label-free detections. SPR sensors have been commercialized for more than two decades, and they represent the current "gold standard" for label-free biosensing. These sensors have been applied in various areas including food quality control, environmental monitoring, drug screening, and early-stage disease diagnosis. SPR sensing is typically conducted using an angular scan [24,25,26,27]: the intensity of reflected light exhibits a dip when the incident angle of light satisfies the SPR excitation condition, and the angular position at which this happens is dependent upon the concentration of analytes near the plasmonic surface. SPR typically takes the form of a broadened Lorentz curve due to optical losses in metallic substrates or nanoparticles. As a result, the quality factor (Q-factor) of such sensors is difficult to reach a value more than 1000. Therefore, SPR-based sensing is not sufficient for the most demanding tasks: (i) Sensing small target analytes with a molecular weight less than 400 Dalton, especially for cancer biomarkers, antibiotics, thyroid hormones, peptides, steroids, and bacterial pathogens in infectious diseases; (ii) detecting biological and chemical molecules with low concentration levels (i.e., < 1 fM or 10^−15^ mol/L) in complex matrices such as urine, saliva, and blood serum. 

Recently, researchers have discovered that the use of optical phase singularity could be a powerful solution to address these two challenges [28,29,30]. Plasmonic resonances typically take the form of broadened Lorentz curves due to optical losses of metallic substrates or nanoparticles. As a result, for the quality factor (Q-factor) of such sensors, it is difficult to reach a value more than 1000. However, plasmonic detection based on phase singularity is not dependent upon angular scanning and not affected by the broad resonance curves [31,32,33]. The key factor that influences phase-related plasmonic sensing is the minimum reflectivity at the resonance angle, which corresponds to a complete optical energy transfer from the incident light to SPR at the sensing interface [34,35]. The challenge of reaching an ultra-high plasmonic sensitivity for detecting small-molecule, low-concentration analytes can be overcome by engineering the sensing substrate to realize the zero-reflection condition. Even if there is a 1.1–1.5-fold increase for the curve widths, it still makes the resolution of the sensors high enough to create a high signal to noise ratio, due to the much more significant phase-related signal change in comparison to angular detection. 

The rapid development of the fabrication techniques for different types of nanomaterials has allowed many breakthroughs in the optical and electronics properties, e.g., high charge carrier mobility, negative refraction, hyperbolic dispersion [36,37,38,39]. Typical examples are the discovery of the monolayer graphene and metamaterial/metasurfaces [40,41], which showed us how the properties and performances of optoelectronic sensing and imaging devices can be improved by engineering the materials at a nanoscale and even, atomic scale. More recently, perovskite nanosheets have been known as a novel optical material with high optical absorption efficiency and could achieve record photovoltaic (PV) power conversion efficiencies (PCEs) up to 24.8% as key units in solar cells devices [42]. They are promising materials for the plasmonic field as the sensing substrate to realize zero-reflection for phase singularity. In 2015, Dou et al. [43] demonstrated the synthesis of atomically thin two-dimensional single crystal perovskite nanosheets ((C_4_H_9_NH_3_)_2_PbBr_4_) by the solution phase growth method. They tuned the thickness of the perovskite layers from a single layer to 3 layers, 8 layers and 22 layers. The corresponding photoluminescence emission peaks are shifted with the changing band gap for these thin perovskite layers. Later, Bao et al. [44] reported the fabrication of the atomically thin 2D CH_3_NH_3_PbX_3_ (X = Cl, Br, or I) perovskite with broadband photoluminescence emission ranging from 660 to 730 nm and a high photoresponsivity of 22 AW^−1^ under the excitation of 532 nm laser sources. 

In this paper, we have designed ultrasensitive plasmonic biosensors with the integration of atomically thin perovskite nanomaterials on metasurfaces reaching a detection limit up to 10^−10^ refractive index units (RIU) for the target sample solutions. More importantly, we have improved the phase singularity detection with the Goos–Hänchen (GH) effect. The GH shift is known to be closely related to optical phase signal changes; it is much more sensitive and more sharp than the phase signal in the plasmonic condition, while the experimental measurement setup is much more compact than that of the common used interferometer scheme to exact the phase signals. Here, we have demonstrated plasmonic sensors with an ultra-high value of 1.2862 × 10^9^ µm/RIU with the optimum configurations for the plasmonic metasensors. For the first time, GH shift generated through the phase singularity based on the 2D perovskite materials is more than three orders of magnitude compared to other metamaterials schemes, including Ag/TiO_2_ hyperbolic multilayer metamaterial (HMM) [45], a metal–insulator–metal (MIM) multilayer waveguide with plasmon-induced transparency (PIT) [46], a metasurface device with a rapid phase gradient [47] and even an electrically controllable graphene nanostructures [48]. The plasmonic GH shift that is reported in this paper is more than six orders of magnitude higher with the atomically thin perovskite metasurfaces (1.2862 × 10^9^ µm/RIU) than those without them (918.9167 µm/RIU). 

## 2. Methods

**Figure 1:**
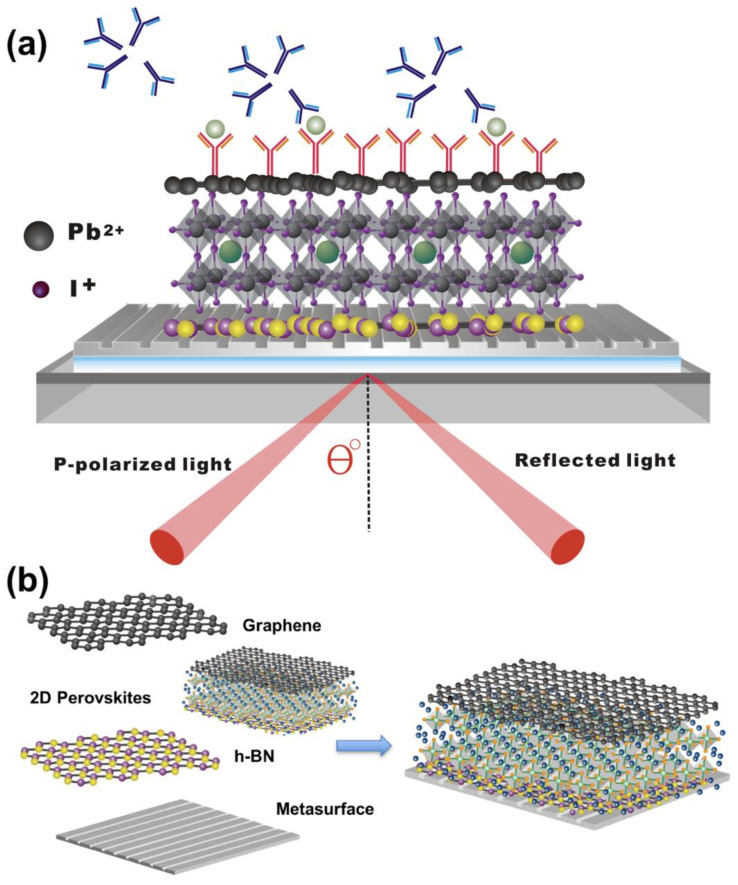
(**a**) Schematic figure of a 2D perovskite-based Goos–Hänchen enhanced surface plasmon resonance (GH-SPR) biosensor, which is integrated with metasurface patterns; (**b**) graphene/2D perovskite/hBN engineered on metasurface patterns.

To evaluate the sensing performances of the 2D perovskite-enhanced plasmonic configurations, we have chosen an established Kretschmann excitation method as the standard modeling of the sensor head, as shown in Figure 1. One advantage of the sensing interface is the sensing substrate; silver thin film is selected rather than a gold one due to the protection of the hexagonal boron nitride (hBN) layer engineered on it. Single layer hBN is also known as white graphene. It has been well demonstrated that the silver substrate could provide a much narrower width of the resonance curves and also deeper resonance dips [49,50]. Two-dimensional perovskite is sandwiched between the hBN and graphene layers, where its optical property will not be affected due to oxidization and liquid contaminations [51,52]. The single graphene layer [53,54] is on top of the configuration to enhance the adsorption efficiency of the targeted biological molecules through pi-stacking forces. For the experimental measurement, reflectance is collected as the intensity ratio between the reflected light from the sensing surface and the incident light. A He–Ne laser (Thorlab Inc., Newton, NJ, USA) was employed to excite the surface plasmon resonance phenomenon at an appropriate incident angle by rotating the translation stage. On the stage, the sensing substrate is attached to the face of prism by optical matching oil (Cargille Labs, Cedar Grove, NJ, USA). A pin hole is added to eliminate the stray light and a high-precision optical power meter (Newport 2832C) is used to monitor light intensity. For phase interrogation, it is known that TM-polarized light suffers a sharp phase change as long as plasmon resonance is excited. Thus, we detect the interference intensity between Transverse magnetic (TM)-polarized light and transverse electric (TE)-polarized light. The incident light is separated into TM-polarized light and TE-polarized light by a polarized beam splitter (PBS). The TM light beam can excite resonance at the sensing surface by the rotation the translation stage at an appropriate incident angle. The TE light beam is modulated by a galvo-mirror (Thorlabs, GVS001). The optical path difference of TE light causes a change in the phase. The TE light interferes with TM light after a PBS and a polarizer. Here, TM light carries the information of the injected sample attached on the sensing surface. The light intensity detected by a photodetector is collected by a data acquisition (DAQ) card (NI PCI-6115, Austin, TX, USA) with a sampling rate of 100 kHz. The differential phase between TM light and TE light can be extracted through Matlab processing procedures. For Goos–Hänchen (GH) interrogation, when plasmon resonance is excited at the metal/dielectric interface, TM light experiences a large GH shift, while TE light does not. Therefore, differential GH lateral position changes between TM light and TE light can show the information of the sample solutions with a low noise. Similarly, PBS separates the incident light beam into TM and TE light. Chopper ensures that only TE light or TM light can get through at a time. The lateral effect position sensor (Thorlabs, PDP90A) is used to detect the beam position of the reflected light from the plasmonic sensor. As a result, the beam position of alternating TM light and TE light is collected by a data acquisition (DAQ) card. The GH shift between TM light and TE light can be obtained after Matlab processing procedures. We will firstly do the experimental measurement with our theoretical results with glycerin for calibration. If the calibration results have matched well with our simulation, we will do the optical biosensing tests with the target analytes.

### 2.1. Optical Parameters of the Sensing Configuration

In this prism-coupling method for plasmonic excitation, we used a high refractive index equilateral SF11 prism. The refractive index of the prism is obtained by the equation below [55]:

(1) $$n_{\mathrm{prism}}={\left(\frac{1.73759695\lambda_{\mathrm{inc}}^{2}}{\lambda_{\mathrm{inc}}^{2}-0.013188707}+\frac{0.313747346\lambda_{\mathrm{inc}}^{2}}{\lambda_{\mathrm{inc}}^{2}-0.0623068142}+\frac{1.89878101\lambda_{\mathrm{inc}}^{2}}{\lambda_{\mathrm{inc}}^{2}-155.23629}+1\right)}^{1/2}$$

where the unit for the incident wavelength is micrometer (μm), and this equation is valid for all the wavelengths in visible and near-infrared regions. The parameters for silver thin film substrate and the titanium adhesion layer of the modeling correspond to the experimental measurement ones that are reported by Palik et al. in reference [56]. The thickness of the titanium layer is 2.5 nm, which is a standard thickness for adhesion layers in conventional SPR experiments. The dielectric constant and thickness of monolayer hBN is obtained according to measurement data by Golla et al. [57] and fixed to be 0.34 nm, which is the same as graphene [58,59]. The optical constant of single layer graphene in the visible region is known to follow the equation below [60,61]:

(2) $$n_{\mathrm{graphene}}=n_{g}+{\mathrm{ik}}_{g},\;\mathrm{where}\;n_{g}=3.0,\;k_{g}=C_{1}\lambda_{\mathrm{inc}}/3,\;C_{1}=5.446/\mu m$$

The dielectric constants of perovskites that are used for the plasmonic modeling extracted from experimental results are shown in Table 1. Here, the change in the refractive index corresponding to the target molecular binding was assumed to be ∆n_bio_, ranging from 10^−8^ refractive index units (RIU) to 10^−1^ RIU [5,62,63]. This range is more than sufficient to reach the detection limit of individual nucleobases’ binding of the single-stranded DNA (ss-DNA). 

### 2.2. Phase (ϕ_p_) and Goos–Hänchen (GH) Shift (L_shift_)

It is known that for conventional surface plasmon resonance (SPR) sensors, detection merits are mainly based on the scanning of the change in reflected light intensity (∆R_SPR_), or the change in resonance angle (∆θ_SPR_) and the change in resonance wavelength (∆λ_SPR_). However, these detection methods are not able to achieve the sensitivity for tiny refractive index changes below 10^−5^ refractive index units (RIU). Recently, the phase interrogation approach has been exploited to significantly improve the sensitivity of the SPR sensors. Experimental schemes for differential phase measurements based on interferometric and ellipsometric configurations have been demonstrated to extract the phase signal (ϕ_p_) of the reflected light. The phase signal led to a much sharper change to the surrounding media at the resonance interface in comparison to the change in the other parameters of reflected light mentioned above. Here, the Goos–Hänchen (GH) shift (L_shift_) emerged as a higher order signal change of the phase, and corresponds to the lateral shift of the reflected beam at the sensing substrate. This lateral shift in the direction along the propagation direction of the surface plasmon waves (SPWs) is similar to the Goos–Hänchen (GH) shift (L_shift_) that is usually observed when an abrupt phase change is induced for phenomena such as the Berry phase, Brewster’s angle phenomenon and the Aharonov–Bohm effect. However, the quantitative measurements of the Goos–Hänchen (GH) shift (L_shift_) for these phenomena are challenging due to the few micrometers magnitude shift in general. Under strong surface plasmon resonance effects, this shift could be optimized and enhanced to 2 orders of magnitude larger. 

We have used the Transfer Matrix method (TMM) with Fresnel equations to do systematic investigation on our 2D perovskite-enhanced plasmonic models. The tangential part of the electromagnetic field for the sensing substrate from the 1st layer to the last layer are followed the boundary condition, as below:

(3) $$\left[\begin{array}{c} E_{1}^{T}\\ H_{1}^{T} \end{array}\right]=M\cdot \left[\begin{array}{c} E_{N-1}^{T}\\ H_{N-1}^{T} \end{array}\right]$$

For the p-polarization light, the Transfer Matrix (TM) for a multilayer model is described as:

(4) $$M=\left[\begin{array}{cc} M_{11} & M_{12}\\ M_{21} & M_{22} \end{array}\right]=\overset{N-1}{\underset{m=2}{\prod }}\left[\begin{array}{cc} \cos\beta_{m} & -\frac{i\left(\sin\beta_{m}\right)}{q_{m}}\\ -i\left(\sin\beta_{m}\right)\cdot q_{m} & \cos\beta_{m} \end{array}\right]$$

(5) $$q_{m}=\frac{\sqrt{\varepsilon_{m}-n_{1}^{2}sin^{2}\theta_{inc}}}{\varepsilon_{m}}\;$$

and here

(6) $$\beta_{m}=\frac{2\pi d_{m}}{\lambda_{0}}\sqrt{\varepsilon_{m}-n_{1}^{2}sin^{2}\theta_{inc}}$$

$\theta_{inc}$ in Equations (3) and (4) is the angle of incident with the refractive index of the prism of $n_{1}$.

The Fresnel reflection coefficient for p-polarized light is calculated by:

(7) $$r_{p}=\frac{\left(M_{11}+M_{12}q_{N}\right)q_{1}-\left(M_{21}+M_{22}q_{N}\right)}{\left(M_{11}+M_{12}q_{N}\right)q_{1}+\left(M_{21}+M_{22}q_{N}\right)}\;$$

Thus, the phase ($\phi_{p}$) and Goos–Hänchen (GH) shift (*L_shift_*) of this multilayer model are obtained as [46]:

(8) $$\phi_{p}=\arg\left(r_{p}\right)$$

and

(9) $$L_{shift}=-\frac{\lambda_{inc}}{2\pi \sqrt{\varepsilon_{1}}}\;\frac{\Delta \phi_{p}}{\Delta \theta_{inc}}$$

When the surface plasmon resonance is excited, the value of the reflected light intensity is close to zero, and a large phase jump is observed at the SPR angle. The phase retardation at this singular point leads to a large Goos–Hänchen (GH) shift (*L_shift_*). The phase (*ϕ_p_*) and Goos–Hänchen (GH) shift (*L_shift_*) are very sensitive to the refractive index change $\Delta n_{target}$ of the target sample solutions interacting at the sensing interface. 

The SPR sensitivity based on Goos–Hänchen (GH) shift is defined as [9]:

(10) $$S_{GH}=\frac{\Delta L_{shift}}{\Delta n_{target}}$$

### 2.3. Further Design of the 2D Perovskite-Based Plasmonic Metasurfaces

The design of the metasurfaces are studied based on the effective medium theory (EMT) method. It is known to be used for the optimization of plasmonic nanorod arrays and groove structures. Here, we can obtain the dielectric constant for the periodical array structures respectively for the metasurfaces along the transverse (*ε*_⊥_) and longitudinal directions (*ε*_∥_), as below:

(11) $$\varepsilon_{⊥}=\frac{\left(1+N\right)\varepsilon_{metal}\varepsilon_{dielectric}+\left(1-N\right)\varepsilon_{dielectric}^{2}}{\left(1+N\right)\varepsilon_{dielectric}+\left(1-N\right)\varepsilon_{metal}}$$

(12) $$\varepsilon_{∥}=N\varepsilon_{metal}+\left(1-N\right)\varepsilon_{dielectric}$$

where *N* = 1 – *W_m_*/*P_m_* is the plasmonic concentration for the metallic nanomaterials inside the structures, and *W_m_* and *P_m_* are widths and periodicity of the metasurface nanostructure. This equation is applicable for both metal array and groove designs [68]. In addition to the TMM approach, we also employed the Lumerical Finite-difference time-domain method (FDTD) solutions to carry out the numerical analyses on the electric field distribution under the resonance conditions. 

## 3. Results and Discussion

**Figure 2:**
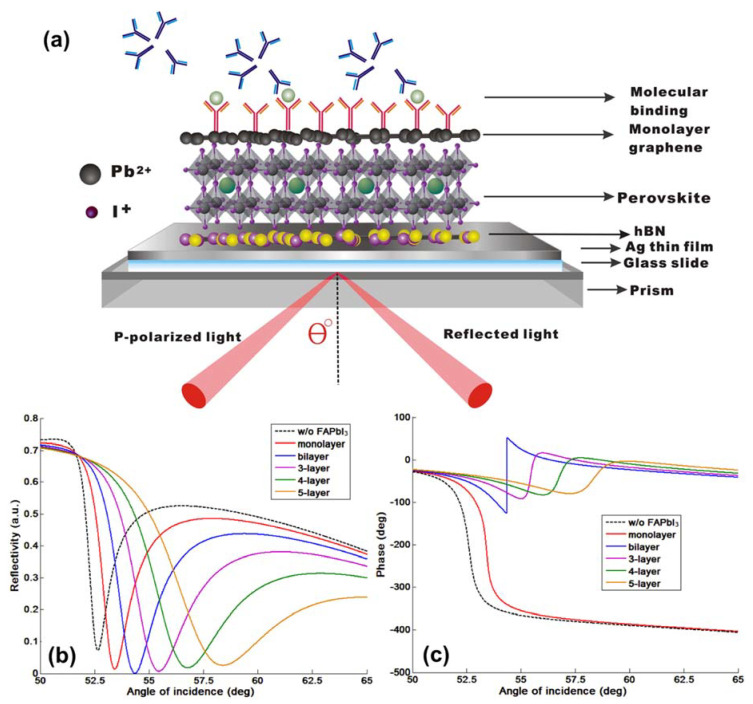
(**a**) Schematic figure of 2D perovskite-based GH-SPR biosensor with continuous silver substrate. SPR curves with reflectivity (**b**) and phase (**c**) signal changes with different numbers of layers of 2D perovskites (FAPbI_3_) engineered on top of the continuous silver substrate at 604 nm.

**Figure 3:**
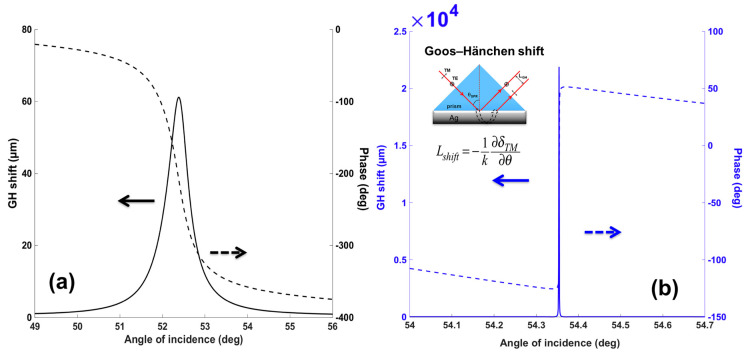
Comparison of Goos–Hänchen (GH) and phase signal changes with silver sensing substrate (**a**) without 2D perovskite, and (**b**) with optimized bilayer 2D perovskites (FAPbI_3_).

Based on Equation (10), the SPR sensitivity based on Goos–Hänchen (GH) shift for the plasmonic metasurface structures is strongly related to the lateral shift of the p-polarization light (L_shift_) for the refractive index change (∆n_target_) induced by the target sample solutions at the sensing interface. One of the prominent physical features for surface plasmon resonance (SPR) is that under the resonance condition, the electric field excited at the substrate would be largely enhanced due to the resonant coupling of the light energy. The maximum electric field intensity can be two orders of magnitude higher than the initial field value of the incident light. As a result, the energy level dissipated to the dielectric layer is correspondingly larger than the total internal reflection (TIR) phenomena with only dielectricity and without the use of the metallic thin films. The resonance energy of the evanescent field penetrating to the surrounding media led to a much increased lateral shift from a few micrometers less than 10 µm (<10 µm) for normal TIR to more than 60 µm (>60 µm) for plasmonic resonance. The Goos–Hänchen (GH) shift can be quantitatively calculated from Equation (9) and is proportional to the phase retardation (∆ϕ_p_), occurring at the sensing substrate. In Figure 2, we have plotted the intensity and optical phase change for the reflected light in the p-polarized direction for the continuous silver film engineered with 2D perovskite layers. We can see that the zero reflection under the resonance coupling corresponds to a phase singularity once, where the lower value of the reflected light led to a sharper phase signal change. The phase singularity and the rapid phase change resulted in a large GH shift as shown in Figure 3. The optical phase change is continuous and every circle is within a phase difference of 2π. Thus, the closer to the singular point of resonance through the sensing configuration, the higher the lateral GH shift could be obtained. 

**Figure 4:**
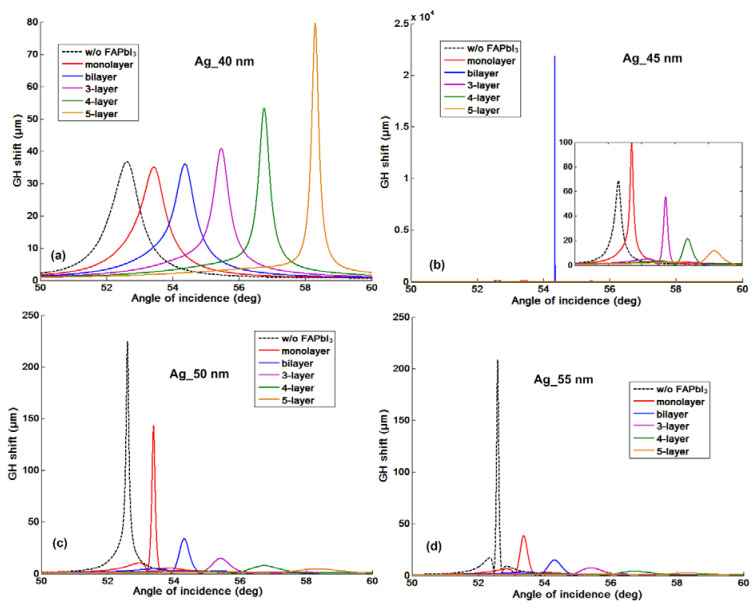
Goos–Hänchen (GH)-SPR signal changes for four different silver substrate thickness ranging from (**a**) 40, (**b**) 45, (**c**) 50 to (**d**) 55 nm, which are respectively tuned by different number of 2D perovskites (FAPbI_3_) layer.

**Figure 5:**
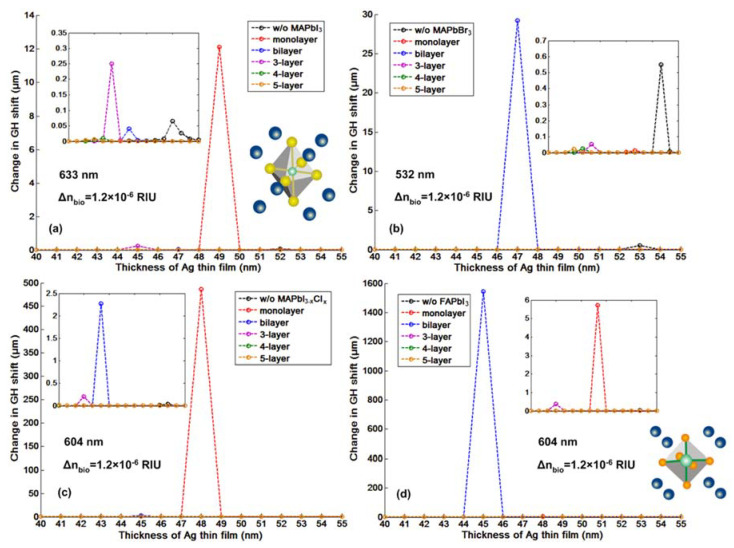
Goos–Hänchen (GH)-SPR signal changes enhanced by four different types of 2D perovskites ranging from (**a**) MAPbI_3_, (**b**) MAPbBr_3_, (**c**) MAPbI_3−x_Cl_x_ to (**d**) FAPbI_3_, which are respectively tuned by different silver substrate thickness. The refractive index change for the targeted sample solutions is fixed to a narrow change of 10^−6^ RIU.

**Figure 6:**
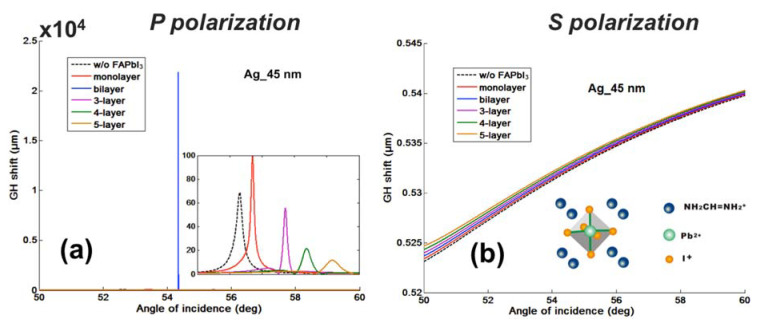
Comparison of the Goos–Hänchen (GH)-SPR signal changes with 2D perovskites (FAPbI_3_) layers under (**a**) p- and (**b**) s-polarization light excitation.

**Figure 7:**
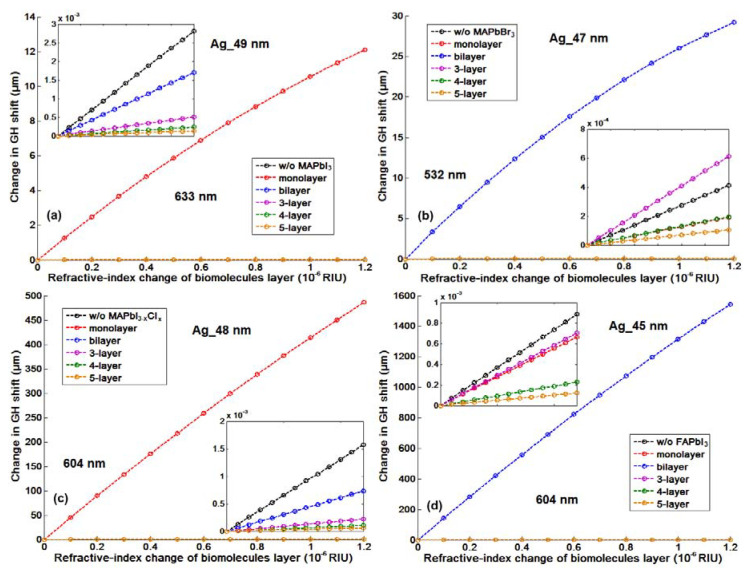
The linear Goos–Hänchen (GH)-SPR signal changes for sensing the target biomolecular binding enhanced by four different types of 2D perovskites: (**a**) MAPbI_3_, (**b**) MAPbBr_3_, (**c**) MAPbI_3−x_Cl_x_ to (**d**) FAPbI_3_, with optimized silver substrate thickness and excitation wavelengths.

**Figure 8:**
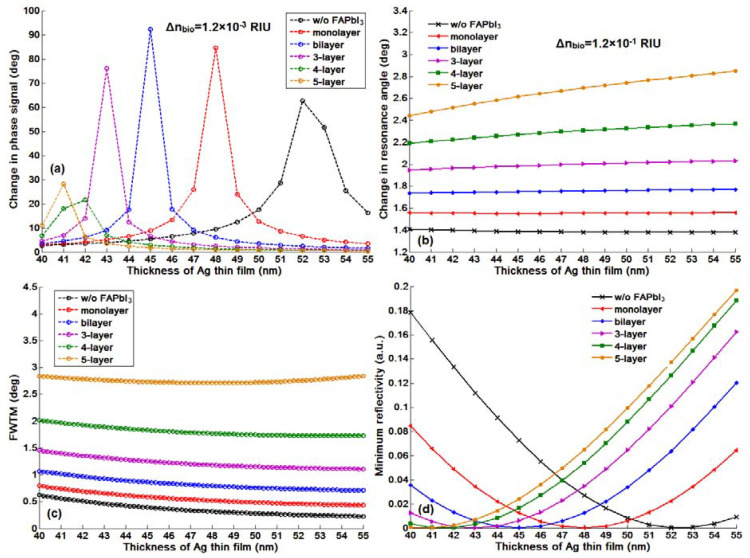
SPR signal changes for 2D perovskites (FAPbI_3_) layer, in addition to the Goos–Hänchen (GH) shift: (**a**) optical phase; (**b**) resonance angle; (**c**) curve width; (**d**) minimum value of the reflectivity at the resonance angle.

As we have discussed in the introduction part, the significant advantage for the phase and GH-SPR detection is that the signal enhancement is higher enough to balance the plasmonic loss induced in the system. Since the 2D perovskite has a higher absorption rate than the silver and gold layers in the visible region (see Figure S1), resonance damping can be tuned to be more precise and more efficient inside the plasmonic sensing structures. With the optimization of the perovskite structures, we can achieve a three orders of magnitude higher GH shift in comparison to that of pure silver thin films (Figure 3b). Firstly, we have systematically studied the plasmonic features with the number of 2D perovskite layers and silver thickness on the GH signal changes of the SPR resonance spectra through Equations (8) and (9). The target analyte molecular interaction that we chose here for detection is mimicking the process of the single stranded DNA (ssDNA) binding to the complementary ones at the interface. The refractive index change induced by the immobilization of the 23-mer ssDNA to form the double-stranded DNA (dsDNA) sequence with picomolar (10^−12^), picomoles/liter was fixed to be ∆n_bio_ = 1.2 × 10^−6^ RIU. Here, we have observed several consistent characteristics for the 2D perovskite-based plasmonic sensing response curves (Figure 2a,b and Figure 3a,b). Firstly, the plasmonic resonance dip angle would redshift to the larger degree with more perovskite layers. This is due to the high value of relative permittivity (i.e., dielectric constant) of the perovskite layers (see Table 1) and is not affected by their imaginary part of these absorption films on the silver substrate. Under the same excitation condition, the positive and large in the value of the real part of the dielectric constant of perovskite would result in a larger surface plasmon wavevector and thus, a lower propagation velocity of the surface plasmon waves. The second optical characteristic for the SPR curve is the widening of the full width at half maximum (FWHM), which is attributed to the imaginary part of the perovskite layers. The large value of their imaginary parts represent a high absorption in the visible region. This enables the flexible tuning of the resonance damping by changing the type of the perovskite and also the precise control of the number of these atomically thin perovskite materials, as shown in Figure 4. Thirdly, the minimal values of the reflected light are changing with the number of the perovskite layers and the thickness of silver thin film in Figure 5, Figure 6, Figure 7 and Figure 8d. The light absorbed by the pure silver substrate is not enough to generate a zero-reflection. This zero-reflection is the key point for achieving optical phase singularity and then, the much more prominent lateral GH shift at the sensing interface (see Figure 8d). More importantly, the thickness of the 2D perovskite is in the range of a few nanometer scale and thus, the width of the resonance curves would not be significantly increased. The detection resolution of the device is not compromised in comparison to the enhanced detection sensitivity. In addition, the GH shift only occurred for the p-polarization light (transverse magnetic waves), which is parallel to plane of incident light as the electrons on the metallic surface were collectively oscillating in this direction and generated plasmon resonance. The s-polarization light (transverse electric waves) remains unaffected, and thus, could be used as a reference control signal during the experimental measurements to avoid the environmental noises, as shown in Figure 6a,b. Through this approach, the signal to noise ratio for the GH setup can be significantly improved with only one signal channel detection. In Figure 2b and Figure 8d, the minimum reflectivity for the bi-layer of FAPbI_3_ perovskite engineered on the 45 nm thick silver substrate is 2.1674 × 10^−8^, where the values are 7.2356 × 10^−2^ and 8.1485 × 10^−3^ for the 45 nm and even 50 nm pure silver thin films. A much sharper phase signal change in a Heaviside manner was induced at the deeper resonance dip. Based on this dramatic phase jump, an enhanced GH shift is generated correspondingly at these resonance angles, as illustrated in Figure 4b. The largest GH shifts for MAPbI_3_, MAPbBr_3_ and MAPbI_3−x_Cl_x_ perovskite-enhanced plasmonic configuration are respectively with 49 nm Ag/monolayer perovskite for 633 nm excitation wavelength (red curve in Figure 5a), 47 nm Ag/bi-layer perovskite for 532 nm excitation wavelength (blue curve in Figure 5b), and 48 nm Ag/monolayer perovskite for 604 nm excitation wavelength (red curve in Figure 5c). The detailed values for the obtained minimum reflectivities under the five different excitation wavelengths and also the GH shifts for each type of perovskite structures are listed in Table 2 and Table 3. For the optimum configurations based on all the calculation results, the highest sensitivity of 1.2862 × 10^9^ µm/RIU at a resonance dip angle of 54.3543° was 49 nm Ag/bi-layer perovskite for 604 nm excitation wavelength (blue curve in Figure 5d). 

It is worth noting that in comparison to the conventional angular scanning and even phase interrogation approach, the GH detection method based on 2D perovskite layer showed a very prominent signal change for the same refractive index change of the target sample solutions. One can compare the maximum phase signal change (∆ϕ_p_ = 92.2749°) and the resonance dip angle shift (∆θ_SPR_ = 1.7484°) from Figure 8a,b induced by the same amount of the target analytes in a large value of 1.2 × 10^−3^ RIU and 1.2 × 10^−1^ RIU with the GH shift change (∆L_shift_ = 1543.5 µm) in Figure 7b and Table 3 for ∆n_bio_ = 1.2 × 10^−6^ RIU. The resonance dip angle shift increases with the increased perovskite thickness and is not affected by the resonance depth during the sensing process. However, the slightly improved angular SPR sensitivity is significantly compromised by the much more wide plasmonic resonance curves. Figure 8c showed that even the full width tenth maximums (FWTMs) for the 4 layer and 5 layer of perovskite thin films largely increased from 0.8655° for the bi-layer to 1.8302° and 2.7320°, respectively. These broadened curve widths would deteriorate the detection resolution of the sensing device due to the inaccuracy of fixing the incident angle to be exactly at the position of the resonance dip (see the orange curves in Figure 1b). Even for the silver thickness of 55 nm and 5 layer of 2D perovskite in Figure 8b, the resonance dip angle only shifted with a small value of 2.6143° for the sample solutions induced with 1.2 × 10^−1^ RIU change. For the ∆n_bio_ = 1.2 × 10^−6^ RIU, the highest angular and phase signal changes with 2D FAPbI_3_ perovskite are respectively 0.00001° and 4.6954°. These signal changes are much lower than the device resolution for some of the rotation stages and phase detectors. Aa a result, the conventional angular scanning and phase interrogation methods are not competent for the detecting of the tiny refractive index change up to 10^−6^ RIU in comparison to the large GH shift of ~1600 µm with 2D FAPbI_3_ perovskite-enhanced plasmonic device. For the 2D MAPbI_3−x_Cl_x_ perovskite structures, the highest GH sensitivity is 4.0518 × 10^8^ µm/RIU, with optimum configuration of 48 nm silver thickness and single layer perovskite (see Table 3). We have also listed the minimum reflectivity and the GH shift for all types of perovskite plasmonic sensors in Table 2 and Table 3. One can observe that the largest GH signal change corresponds to lowest minimum reflectivity, where the zero-reflection would trigger the phase singularity and thus, a significant GH shift. 

**Figure 9:**
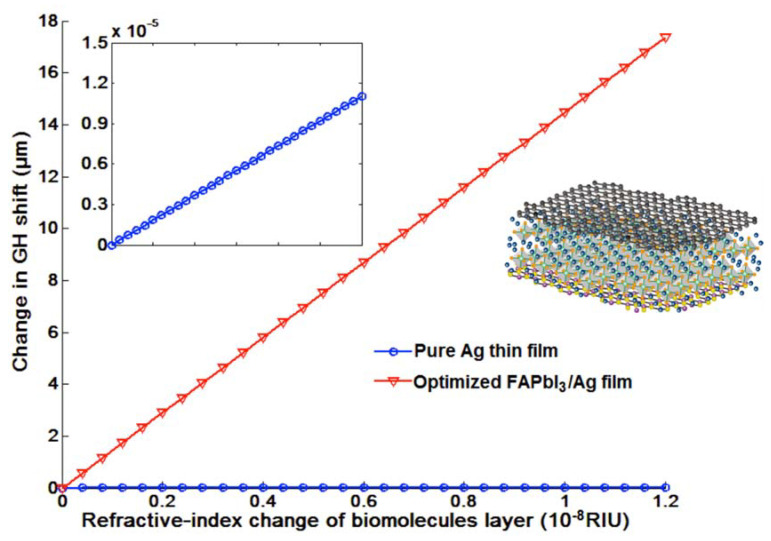
GH sensitivity comparison with (red curve) and without (blue curve) optimized 2D perovskites (FAPbI_3_) layer for a narrow refractive index change up to 10^−8^ RIU.

**Figure 10:**
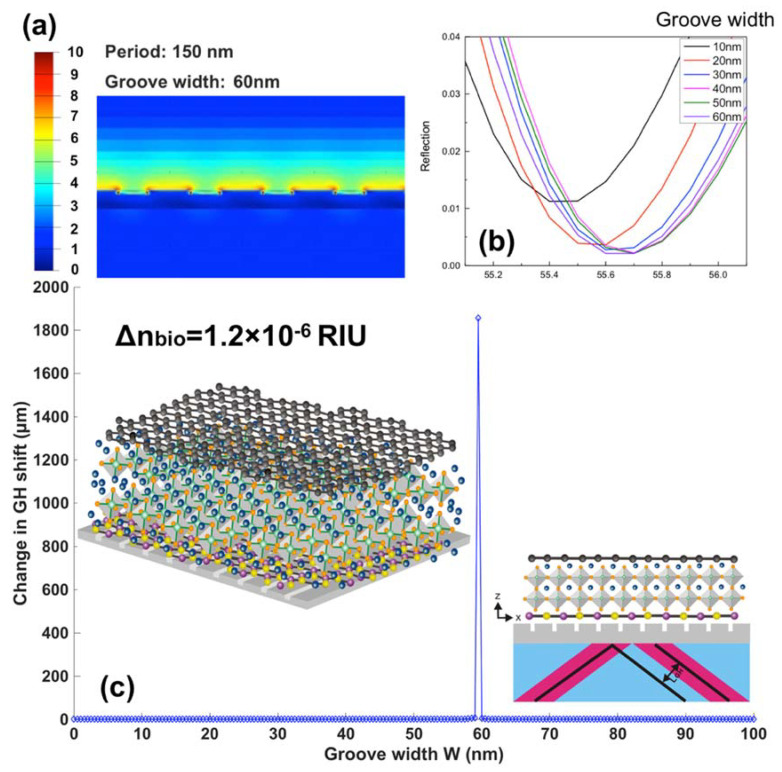
(**a**) Electric field distribution of 2D perovskite-based metasurface patterns under the SPR excitation condition; SPR curves with (**b**) Reflectivity and GH (**c**) signal changes tuned by the groove width of the metasurface structure.

GH shift changes for optimized 2D perovskite plasmonic structures are shown in Figure 7. In addition to the 1543.50 µm for FAPbI_3_ perovskite, there are 12.0978 µm for MAPbI_3_, and 29.2289 µm for MAPbBr_3_ and 486.2172 µm for MAPbI_3−x_Cl_x_ perovskite. These signal changes are much higher than pure graphene-enhanced SPR sensors (single layer graphene engineered on 50 nm silver substrate) with a lateral shift of 0.0028 µm. The capability to detect the small refractive index change promised the sensing of low-concentration chemical and biological molecules up to 1 fM (10^−15^ mol/L) and even 1 aM (10^−18^ mol/L). One can choose the type of perovskite configurations based on the molecular weight and also the concentration range. To achieve wide dynamic detection range, we can use the tunable laser to change the excitation wavelength for continuous sensing of both small and large target analytes with different concentrations. In Figure 9, a linear and large GH shift ∆L_shift_ = 17.3576 µm is shown, corresponding to the refractive index induced by the target biomolecules ∆n_target_ = 1.2 ×10^−8^ RIU. The GH signal ~18 µm was enhanced by six orders of magnitude compared to those of pure silver ones (1.1027 × 10^−5^ µm). Thus then, sensitivity with the atomically thin perovskite metasurfaces is 1.2862 × 10^9^ µm/RIU, which is more than 10^6^ times than 918.9167 µm/RIU for silver sensing substrate with the same thickness. To realize the ultimate goal of single molecular and even submolecular detection, we have also used the effective medium theory (EMT) from Equations (11) and (12), the Lumerical Finite-difference time-domain method (FDTD) for integrating the 2D perovskite into a plasmonic metasurface structure and obtained a much enhanced GH-SPR sensitivity and also localized electric field enhancement. By optimizing the width from 10 to 60 nm of the silver groove structure for a fixed period of 150 nm, the resonance curve could be further damped and more close to the zero-reflection. The depth of the groove is fixed at 3 nm, which is optimized by the FDTD calculation from 0 to 5 nm (see Figure S2). The zoom-in curves at the resonance dips centered around 55.6° were plotted in Figure 10b and the complete reflection curves are shown in Figure S3. With a fixed groove width, the SPR resonance curves would become broadened with increasing groove depth. However, if the groove depth is not large enough, the electric field enhancement is not prominent and also, the molecules length is usually more than 2 nm. Thus, there is a balance between electric field enhancement and the widened resonance curves. As mentioned above, the broad curve would affect the detection resolution, since the incident angle should be fixed at the resonance dip angle before the GH-SPR detection for the target analytes. A 20% larger GH shift of 1854.91 µm was obtained as shown in Figure 10c, corresponding to a higher sensitivity of 1.5458 × 10^9^ µm/RIU. Moreover, the strong electric field between the edge of the groove would further enhance the detection of the binding interaction of the biological molecules (Figure 10a). The electric field is enhanced by 100-fold at the edge corner compared to the intensity values of both the incident light waves and the evanescent field excited on the base substrate. 

## 4. Conclusions

An ultrasensitive plasmonic biosensor has been designed in this paper by engineering the atomically thin 2D perovskite nanostructures on the silver metasurfaces. A record-high GH-SPR sensitivity of 1.2862 × 10^9^ µm/RIU was achieved, which is six orders of magnitude higher than the performance with the conventional continuous silver thin film (918.9167 µm/RIU). The atomically thin perovskite nanomaterials with high absorption rates enable the precise tuning of the depth in the plasmonic resonance dip. We have also provided our comparison plasmonic results for experimental and simulation data for different layers of atomically thin nanomaterials in Figures S4–S6. These results also supported that the optimum configuration can lead to the more enhanced plasmonic sensing signal both for the glycerol calibration tests and for the protein molecular detections. Thus, one can optimize the structure to reach the near zero-reflection at the resonance angle and a sharp phase singularity could be induced, leading to a much more enhanced GH lateral shift at the sensing interface. The 2D perovskite layers were sandwiched between a single layer hexagonal boron nitride (hBN) and graphene to avoid their degradation by water and humidity. The top layer of graphene was used to capture the target biomolecules due to the pi-stacking interaction between the hexagonal carbon ring structures. This advantage could also enhance the adsorption efficiency of the analytes. By integrating the 2D perovskite nanolayer into the metasurface array structure, the GH sensitivity was further improved to 1.5458 × 10^9^ µm/RIU. More importantly, a strong localized electric field enhancement was excited between the groove edge. We believe that this enhanced electric field together with the enhanced GH shift would enable the single molecular or even submolecular detection for hard-to-identify chemical and biological markers, including single nucleotide mismatch in the DNA sequence, toxic heavy metal ions and tumor necrosis factor-α (TNFα).

## Figures and Tables

**Table 1:** The dielectric constants of MAPbI_3_, MAPbBr_3_, MAPbI_3−x_Cl_x_, and FAPbI_3_ perovskites used for the modeling of GH-SPR sensitivity, which are obtained from experimental measurements.

Type of Perovskite	λ = 488 nm	λ = 532 nm	λ = 604 nm	λ = 633 nm
MAPbI_3_ [64]	ε′ = 6.7737,	ε′ = 7.5816,	ε′ = 7.0131,	ε′ = 6.7637,
	ε″ = 3.6584	ε″ = 2.5110	ε″ = 1.3300	ε″ = 1.1484
MAPbBr3 [65]	ε′ = 4.3931,	ε′ = 4.7040,	ε′ = 4.4100,	ε′ = 4.3264,
	ε″ = 0.5460	ε″ = 0.9592	ε″ = 0.0109	ε″ = 0.0083,
MAPbI_3−x_Clx [66]	ε′ = 5.8968,	ε′ = 6.8985,	ε′ = 6.5025,	ε′ = 6.4752,
	ε″ = 3.5926	ε″ = 2.5632	ε″ = 1.6448	ε″ = 1.4336,
FAPbI3 [67]	ε′ = 6.0885,	ε′ = 7.3767,	ε′ = 8.1213,	ε′ = 7.8432,
	ε″ = 5.4468	ε″ = 4.7144	ε″ = 1.9516	ε″ = 1.2926

**Table 2:** List of the parameters for the 2D perovskites with the best GH-SPR sensing performances for the four different excitation wavelengths ranging from 488 to 633 nm.

Excitation Wavelength λ (nm)	Type of P	Silver Thickness (nm)	Optimized P Layers (L)	Min R (a.u.)	Δθ_SPR_ (Deg) (Δn_bio_ = 0.12)	ΔL_shift_ (μm) (Δn_bio_ = 1.2 × 10^−6^)	Sensitivity (μm/RIU)	FWTM (Deg)
488	MAPbI_3−x_Cl_x_	44	1	1.5395 × 10^−7^	2.7973	19.3817	1.6151 × 10^7^	1.9128
532	MAPbBr_3_	47	2	1.5852 × 10^−7^	2.3414	29.2289	2.4357 × 10^7^	1.2922
604	MAPbBr_3_	53	4	9.4180 × 10^−6^	1.8205	10.8690	9.0575 × 10^6^	0.4442
604	FAPbI_3_	45	2	2.1674 × 10^−8^	1.7484	1.5435 × 10^3^	1.2862 × 10^9^	0.8655
633	FAPbI_3_	49	1	7.6131 × 10^−7^	1.3515	30.5218	2.5435 × 10^7^	0.3517

**Table 3:** Summary of the conditions for each type of 2D perovskite to achieve the largest Goos–Hänchen lateral shift while simultaneously with high resolution.

Excitation Wavelength λ (nm)	Type of P	Silver Thickness (nm)	Number of P Layers (L)	Minimum Reflectivity	θ_SPR_ (Deg) w/o Biomolecules	ΔL_shift_ (μm) (Δn_bio_ = 1.2 × 10^−6^)	Sensitivity (μm/RIU)	FWTM (Deg)
633	MAPbI_3_	49	1	1.6230 × 10^−6^	52.8446	12.0978	1.0082 × 10^7^	0.3439
532	MAPbBr_3_	47	2	1.5852 × 10^−7^	55.7627	29.2289	2.4357 × 10^7^	1.2922
604	MAPbI_3−x_Cl_x_	48	1	1.2960 × 10^−7^	53.2981	486.2172	4.0518 × 10^8^	0.5011
604	FAPbI_3_	45	2	2.1674 × 10^−8^	54.3543	1.5435 × 10^3^	1.2862 × 10^9^	0.8655

## Supplementary Materials

The following are available online at https://www.mdpi.com/2079-4991/10/7/1289/s1, Figure S1: Experimental measurement results showing the broad absorption spectra of CH_3_NH_3_PbI_3_ (MAPbI_3_) perovskites, Figure S2: SPR curve widths tuned by the groove depth of 2D Perovskite-based metasurface structure, Figure S3: SPR curves with Reflectivity tuned by the groove width of the metasurface structure with 2D perovskite layers, corresponding to the zoom-in Figure 10b, Figure S4: Experimental and simulation results based on three-layer graphene/gold metasurfaces. Theoretical curves of (a) reflectance with respect to various incident angles in the air, (c) differential phase and (e) differential GH shift between p-polarized light and s-polarized light of glycerol solutions with different concentrations. Experimental curves of (b) reflectance, (d) differential phase and (f) differential GH shift. The resonance angle is 44.10° in good agreement with the theoretical one. The phase and GH shift shows high sensitivity as 22,842 deg/RIU and 7,500 μm/RIU respectively, Figure S5: (a) and (b) Experimental and simulation data, respectively, of GH shift in a device based on a bilayer WS_2_, Figure S6: The averaged GH shift of BSA molecules with molar concentration from 1aM to 1mM based on bilayer WS_2_/gold metasurfaces. The insert curve gives the binding trace of BSA molecules during a short time.
