# Supplementary material for: Plasmonic Metasensors Based on 2D Hybrid Atomically Thin Perovskite Nanomaterials
Source: Nanomaterials (Basel). 2020 Jun 30;10(7):1289. doi: 10.3390/nano10071289 (PMC7407500; doi:10.3390/nano10071289)
Supplement: Supplementary file 1 [file nanomaterials-10-01289-s001.pdf]

nanomaterials

Article

Plasmonic Metasensors based on 2D hybrid atomically thin perovskite nanomaterials

Shuwen Zeng [1,2*†], Guozhen Liang [2†], Alexandre Gheno[1], Sylvain Vedraine[1], Bernard Ratier[1], Ho-Pui Ho[3], Nanfang Yu[2*]

[1] XLIM Research Institute, UMR 7252 CNRS/University of Limoges, 123 Avenue Albert Thomas, Limoges, France;

[2] Department of Applied Physics and Applied Mathematics, Columbia University, New York, USA

[3] Department of Biomedical Engineering, The Chinese University of Hong Kong, Shatin, N.T., Hong Kong;

† †These authors contributed equally to this work.

* Correspondence: shuwen.zeng@unilim.fr (S.Z.), ny2214@columbia.edu (N.Y.)

Received: date; Accepted: date; Published: date

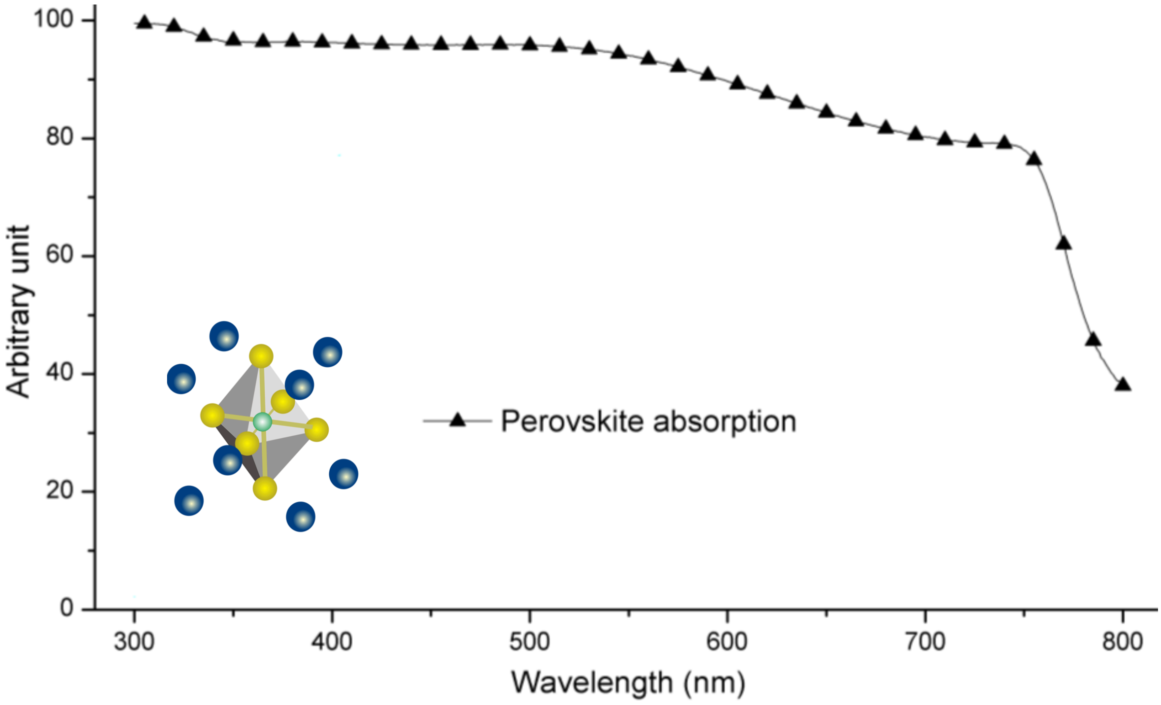

Figure S1. Experimental measurement results showing the broad absorption spectra of $CH_3NH_3PbI_3$ ($MAPbI_3$) perovskites.

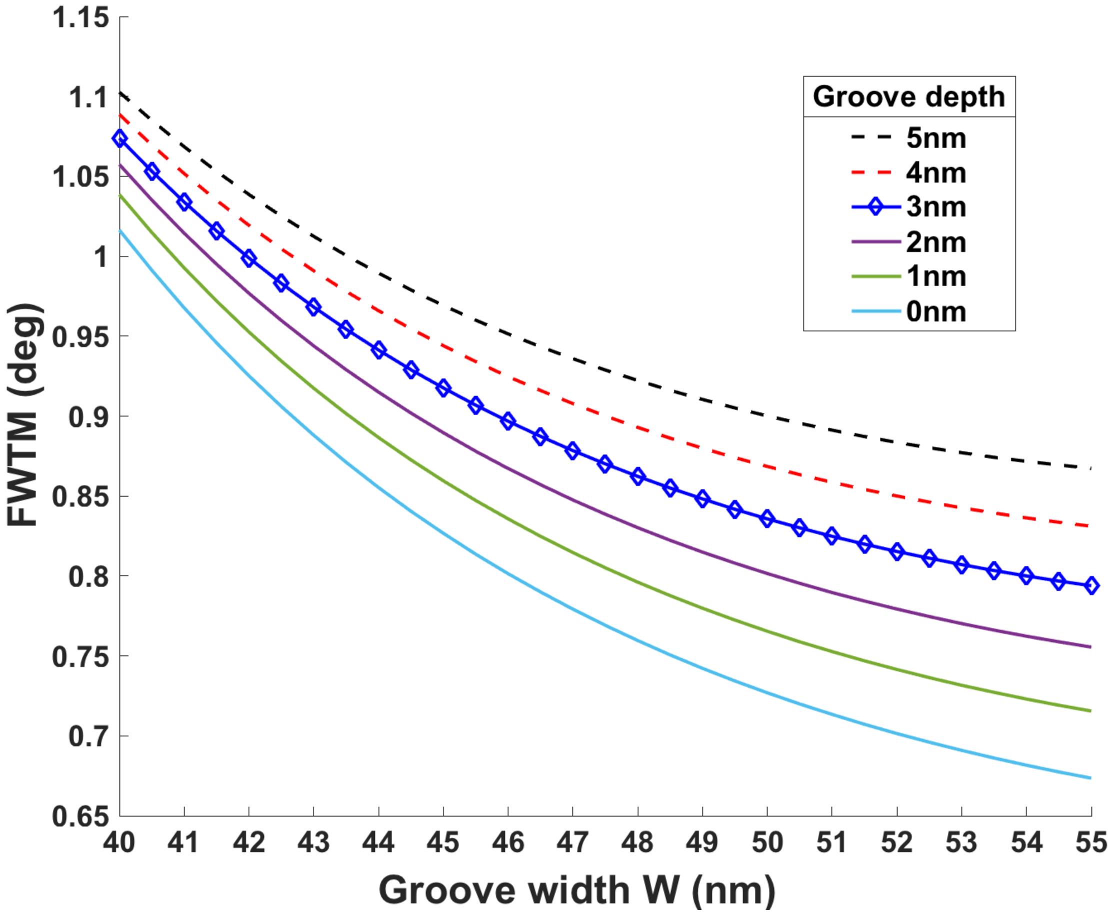

Figure S2. SPR curve widths tuned by the groove depth of 2D Perovskite-based metasurface structure.

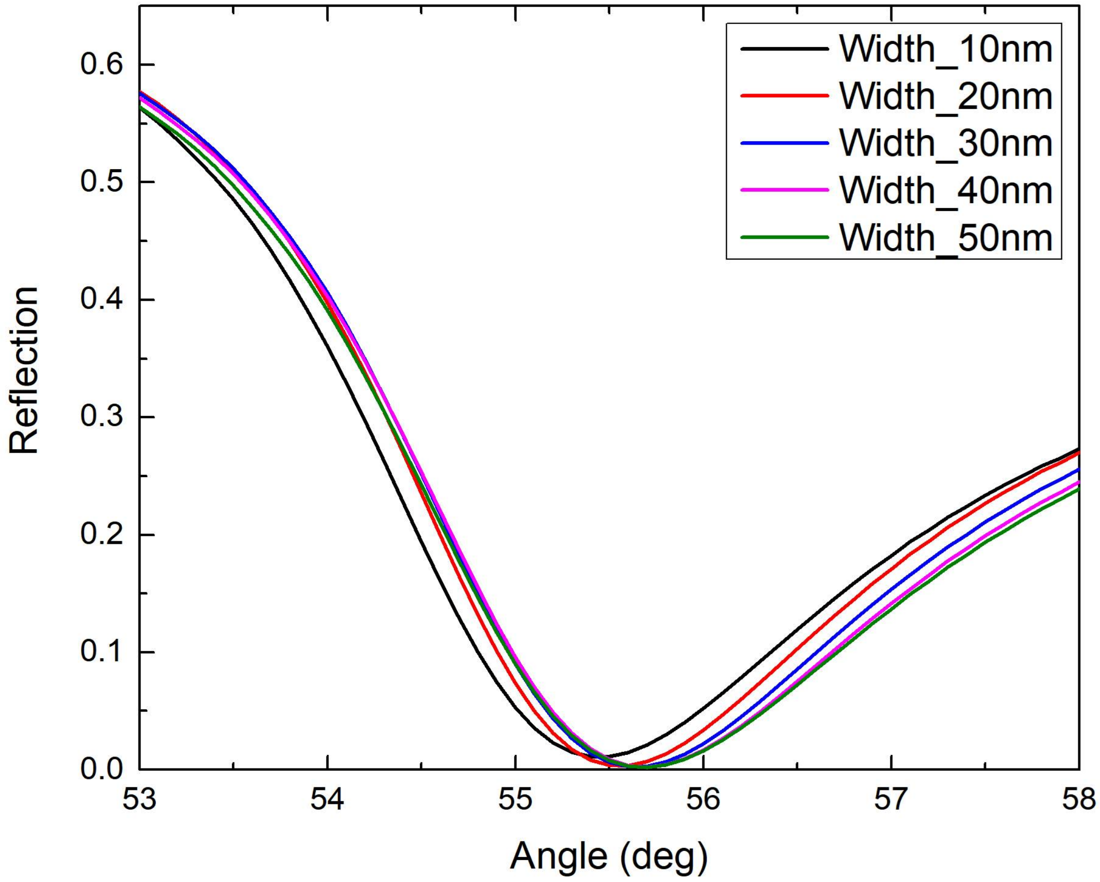

Figure S3. SPR curves with Reflectivity tuned by the groove width of the metasurface structure with 2D perovskite layers, corresponding to the zoom-in Figure 10b.

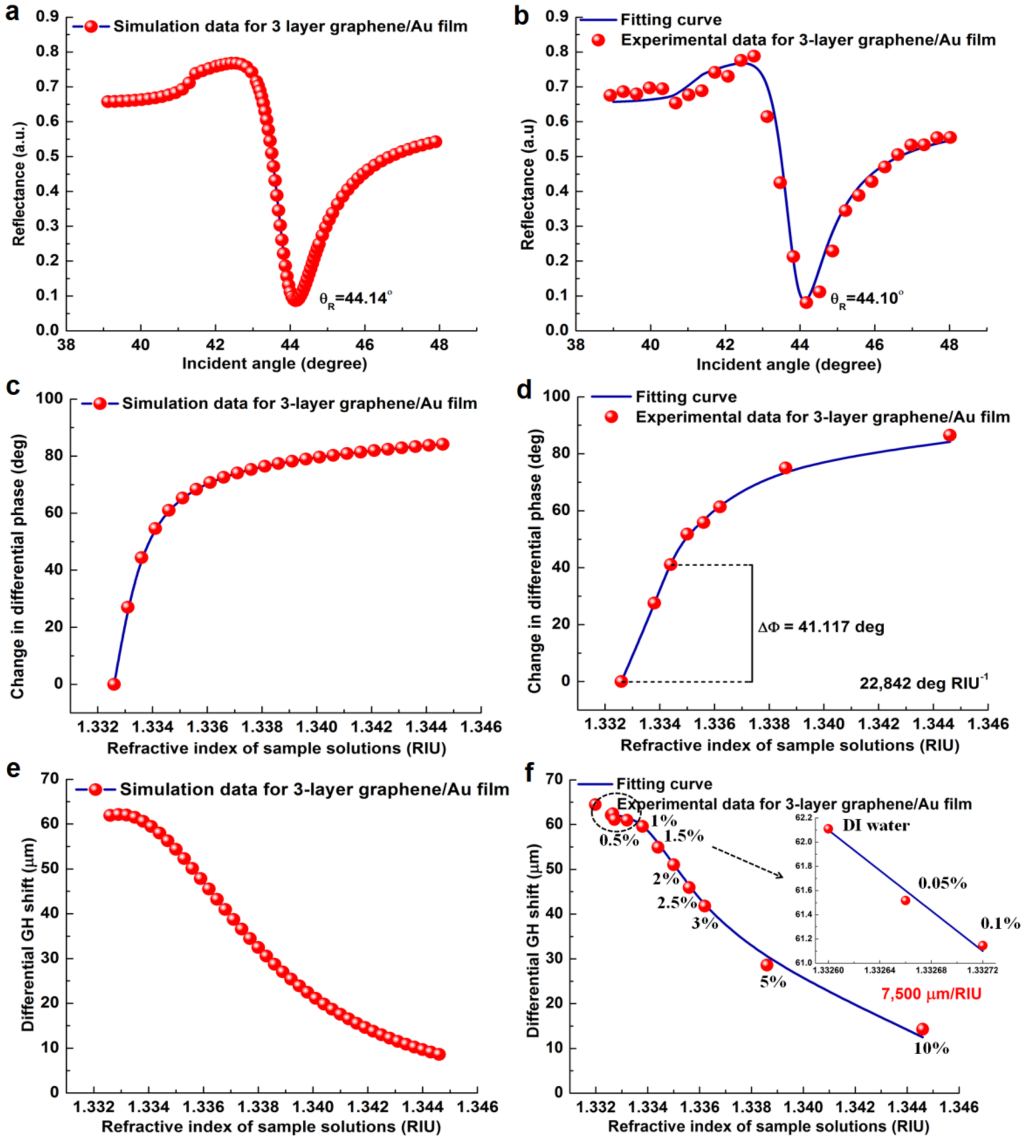

Figure S4. Experimental and simulation results based on three-layer graphene/gold metasurfaces. Theoretical curves of a) reflectance with respect to various incident angles in the air, c) differential phase and e) differential GH shift between p-polarized light and s-polarized light of glycerol solutions with different concentrations. Experimental curves of b) reflectance, d) differential phase and f) differential GH shift. The resonance angle is 44.10° in good agreement with the theoretical one. The phase and GH shift shows high sensitivity as 22,842 deg/RIU and 7,500 μm/RIU respectively.

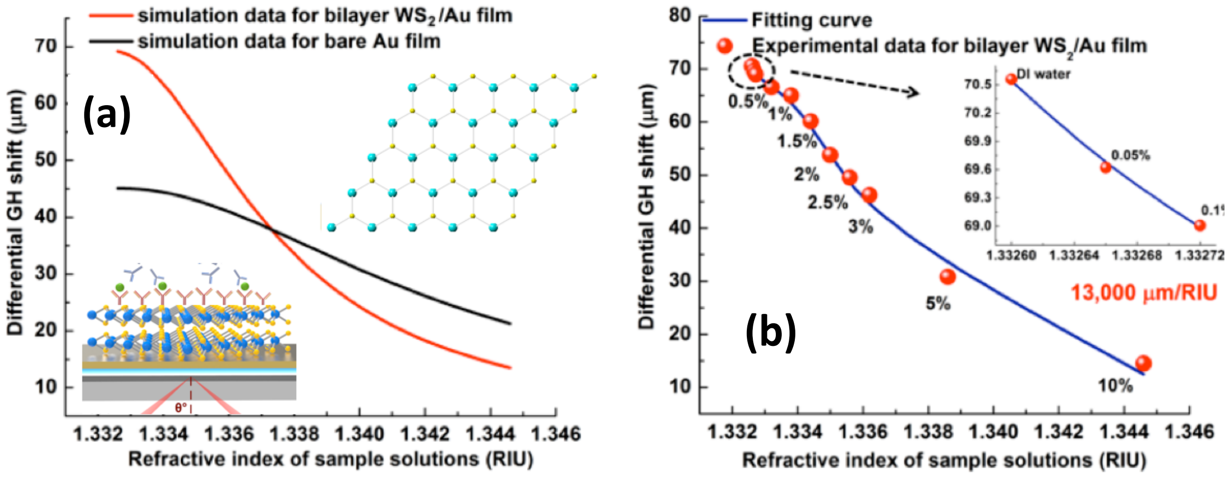

Figure S5. (a) and (b) Experimental and simulation data, respectively, of GH shift in a device based on a bilayer WS_2.

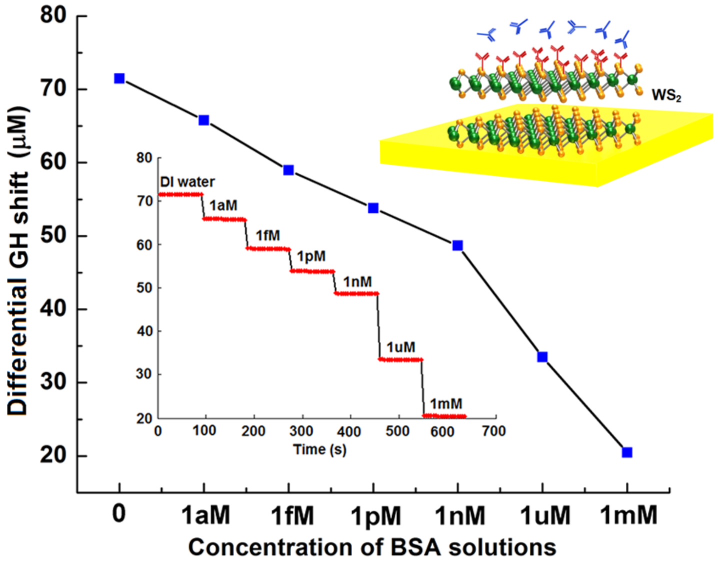

Figure S6. The averaged GH shift of BSA molecules with molar concentration from 1aM to 1mM based on bilayer WS_2/gold metasurfaces. The insert curve gives the binding trace of BSA molecules during a short time.
